# Fooling the Eyes: The Influence of a Sound-Induced Visual Motion Illusion on Eye Movements

**DOI:** 10.1371/journal.pone.0062131

**Published:** 2013-04-26

**Authors:** Alessio Fracasso, Stefano Targher, Massimiliano Zampini, David Melcher

**Affiliations:** 1 Experimental Psychology, Utrecht University, Utrecht, The Netherlands; 2 Center for Mind/Brain Studies and Department of Psychology and Cognitive Sciences, University of Trento, Rovereto, Italy; 3 Center for Mind/Brain Studies and Department of Psychology and Cognitive Sciences, University of Trento, Rovereto, Italy; 4 Center for Mind/Brain Studies and Department of Psychology and Cognitive Sciences, University of Trento, Rovereto, Italy; University of Nevada Las Vegas, United States of America

## Abstract

The question of whether perceptual illusions influence eye movements is critical for the long-standing debate regarding the separation between action and perception. To test the role of auditory context on a visual illusion and on eye movements, we took advantage of the fact that the presence of an auditory cue can successfully modulate illusory motion perception of an otherwise static flickering object (sound-induced visual motion effect). We found that illusory motion perception modulated by an auditory context consistently affected saccadic eye movements. Specifically, the landing positions of saccades performed towards flickering static bars in the periphery were biased in the direction of illusory motion. Moreover, the magnitude of this bias was strongly correlated with the effect size of the perceptual illusion. These results show that both an audio-visual and a purely visual illusion can significantly affect visuo-motor behavior. Our findings are consistent with arguments for a tight link between perception and action in localization tasks.

## Introduction

In a seminal and influential study in neuropsychology [1], it has been suggested that visual information for perception and for action are processed separately through two independent streams [2], [3]. According to this dual visual system hypothesis [1], object recognition is supported by projections from the primary visual cortex to the inferotemporal cortex (ventral stream), while action information is processed in a separate pathway that runs from the primary visual cortex to the posterior parietal cortex (dorsal stream). Crucially, the dual-streams proposal suggests that the two systems rely upon independent neural representations. In support of this proposal, experimental data has been reported in studies of hand movements. For example, it has been shown that uncertainty measures of visually guided grasping (just noticeable differences across length estimates) do not follow the basic Weber law of scaling with stimulus length [4] in sharp contrast with measures of perceptual estimation (but see [5] for an alternative explanation). In further support of this claim, it has been reported that some visual illusions influence perceptual judgments while leaving hand motor responses unaltered [6]. However, the interpretation of such data has been strongly debated [7], [8], [9], [10]. Using different methods which carefully calibrate perceptual measures and motor tasks, it has been shown that visual illusions can affect both perceptual judgments and motor behavior [11], [12]. Moreover, in the case of visual illusions driven by moving stimuli, it has been shown that the pattern of differences between action and perception depend entirely on the stimuli and methods used [13], undercutting much of the evidence which has been taken as support for the dual system proposal.

In addition to studies of grasping and other hand movements, the dual visual system hypothesis has also been tested using eye movement measures. Importantly, saccadic eye movements differ from hand movements in that they are stereotyped and ballistic in nature [14]. Indeed, saccades provide information about visual-spatial representations but also are typically outside of conscious control and reflection (we normally make an eye movement without thinking explicitly about where it will land). For this reasons, saccades can be used to test where the oculomotor system localizes a stimulus and compare this process with more explicit perceptual judgments. Such studies, however, have yielded contradictory interpretations.

Some studies of eye movements have been taken as evidence supporting the dual-streams hypothesis. Near the onset of a ballistic eye movement, the visual perception of the location of briefly presented stimuli in the middle of eyes' trajectory is grossly distorted along with the direction of the movement itself, a phenomenon known as peri-saccadic mislocalization. When the screen darkens right after stimuli presentation (reducing visual cues), rapid pointing remains accurate towards the real physical target position. However, perception measured by verbal reports is distorted by the incoming saccade [15].

In contrast, other studies of eye movements have been taken as evidence against the two visual system hypothesis. In particular, a meta-analysis of the effect of the Muller-Lyer illusion (ML) on eye movements [16] provides evidence against a functional dissociation between visuo-motor and perceptual systems. In this case, the experimental paradigm can influence whether the illusion influences saccadic landing positions or not. It has been shown that the effect of the Muller-Lyer illusion on eye movements is modulated by saccadic latency [17], with longer latency saccades being less influenced by the visual illusion than shorter latency saccades. Moreover, the predictability of target stimuli modulated the effects of the ML on eye movement amplitude, with a stronger illusion effect for unpredictable stimuli locations [17].

Previous studies of visual illusory context effects on perception and action raise the question of whether auditory context will also influence motor behavior. It has been reported that auditory information affects visual motion perception in a variety of ways. For example, ambiguous apparent motion configurations can be strongly biased by the presence of a transient sound which provides temporal capture [18]. Likewise, the presence of a transient auditory stimulus at the point when two visual stimuli move across each other can induce a percept of the stimuli bouncing off of each other rather than crossing their paths [19].

Recently, some studies have shown not only that auditory stimulation modulates perception of a moving stimulus, but can even evoke motion perception for an otherwise static stimulus. One particularly interesting case, which we study here, is the sound-induced visual motion illusion (SIVM: [20]). A flashing, high contrast bar presented at a fixed location in the periphery of the visual field is perceived as shifting in lateral motion when synchronized with an alternating left and right sound. Moreover, a measurable aftereffect can be obtained by this induced motion [21]. One interesting aspect of this illusion, relevant for the current study, is that sound causes a visual-spatial mis-localization, whereas most previous studies of audio-visual illusions have used vision to bias auditory location or audition to bias visual timing [22], [23], [24].

The current study is the first attempt (to our knowledge) to study the influence of an audio-visual illusion on eye movements. The present experiment allows us to test and evaluate the dual-visual systems hypothesis within the cross-modal domain (audio-visual stimulation). With this goal in mind, we took advantage of the aforementioned SIVM [20], testing its effect on saccadic landing positions. Considering the previous work on the influence of visually driven illusions on visuo-motor behaviour [25], [10], we hypothesized that the SIVM illusion would influence oculomotor responses. To further test how closely the perceptual and action systems were linked, we also used inter-subject variability in the illusion magnitude and saccade landing positions to test whether the magnitude of the perceptual illusion correlated with the magnitude of the action (saccade) effect.

Strong independence between perception and visuo-motor systems would predict a measurable SIVM for perception, while leaving visuo-motor behaviour unaffected by the illusion. A weak independence would instead predict an influence of the SIVM on saccades, though with a different pattern than on perceptual measures. In the case of overlapping spatial representations for both the perceptual and visuo-motor systems, we would expect perceptual and motor responses to be similarly affected by the SIVM.

## Materials and Methods

### Participants

Thirteen participants took part in the study. Participants were all students of the University of Trento (7 female, mean age of 25 years; range from 20 to 46 years), reported normal hearing and had normal or corrected-to-normal vision. The experiment was conducted in accordance with the ethical standards laid down in the 1964 Declaration of Helsinki (most recently amended in 2008, Seoul), as well as the ethical guidelines laid down by the University of Trento ethics committee (Comitato etico per la sperimentazione con l'essere umano). All participants were naïve as to the purpose of the experiment. Participants gave their written informed consent to participate in this study. All the experiments were conducted in the laboratories of the Center for Mind/Brain Sciences of the University of Trento in Rovereto, Italy.

### Apparatus and stimuli

Participants sat at a table in a dimly lit room (average luminance 40 cd/m^2^) at a distance of 60 cm from a 22 inch LCD screen (HP Compaq LA 2205 WG at 60 Hz, resolution: 1680×1024) used for presenting the visual stimuli. Participants’ head movements were restrained by an adjustable chin rest. The visual stimulus was a stationary, flickering white vertical bar (10×0.4 degrees of visual angle) shown for a 100 ms duration with an inter-stimulus interval (ISI) of 400 ms. The auditory stimulus consisted in a white noise burst of 75 ms duration presented through headphones (Sony MDR-XD200). On each trial, the bar was presented at one of three different eccentricities (15, 16 or 17 degrees of visual angle) with respect to the fixation point. At the beginning of each trial the fixation point was positioned either to the left or to the right of the display midline at an eccentricity of 4 degrees of visual angle. The fixation point and the flickering bar were always presented on the opposite sides of each other in relation to the display midline. The eccentricity of the bar and the position of the fixation point were varied randomly across trials. The bar might flicker either 5 or 6 times during each trial, in order to prevent participants from easily predicting the end of the trial.

In separate blocks, the addition of the synchronous sound was varied, so that the flickering bars were either shown alone (vision only condition: V) or with the sound (audio-visual condition: AV). The onset of the AV stimuli was synchronized using a digital oscilloscope (Agilent Technologies MSO 6054A).

In AV trials, the sound switched between the right and left ear synchronously with the onset of the flickering bar in order to induce the visual illusion (i.e., the SIVM) in the direction of the sound movement.

**Figure 1:**
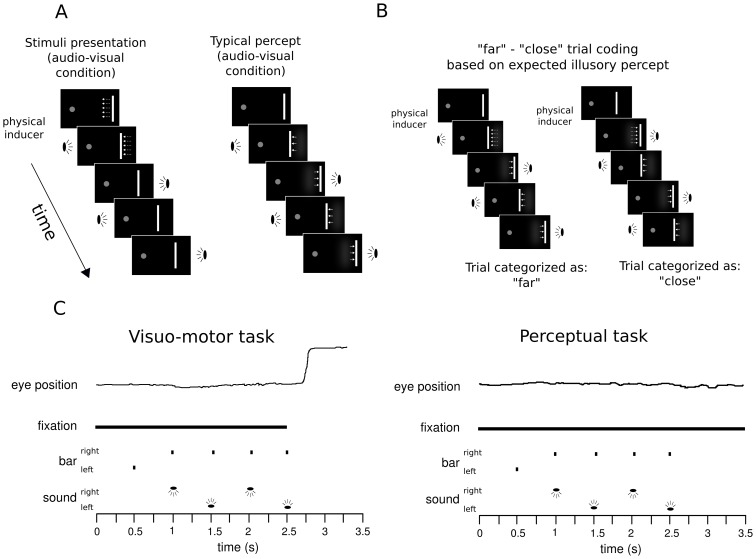
Visual and auditory stimuli used during the experiment. Panel A: stimuli presentation and typical percept for an audio-visual (AV), 5 bar repetition trial; between the first and second bar presentation a physical shift was introduced (see Procedure and Experimental Design), the remaining bars were presented always in the same position. Panel B: “far” and “close” trial coding based on the expected percept. Responses were coded as “far” and “close” as well. During the analysis phase the proportion of “far” responses for the visual-only and audio-visual condition and the expected response were analyzed (see “Data Analysis” section). Panel C: trial procedure for the visuo-motor and perceptual tasks for an audio-visual stimuli condition, along with typical eye movement traces. The visual-only condition was identical except that the sound was not provided. Each bar was presented for 100 ms with an ISI of 400 ms. In the visuo-motor task participants were instructed to perform an eye movement after the fixation point disappeared (50 ms before the last bar offset). In the perceptual task participants reported the perceived direction of the last bar (leftward vs. rightward).

However, a preliminary pilot study revealed that although the sound did induce perceived motion, there was not a clear one-to-one matching between sound direction and bar illusory motion direction. That is, on the majority (∼65%) of the trials a left-to-right sound direction could induce a coherent left to right bar motion, while on other trials the same left-to-right sound could induce an opposite right-to-left bar motion. This randomly alternating direction of the illusion could not be controlled *a priori* and thus would have posed a serious problem in the analysis phase. In our experiment, unlike earlier studies of the SIVM, the direction of the illusory motion was as important as whether or not it occurred. To overcome this problem, a physical displacement of the bar (henceforth, “physical inducer”) was presented at the start of each trial to match the direction of the apparent motion of the sound. More precisely, from the first to the second bar onset, the spatial position of the bar was physically displaced by 1 degree of visual angle towards the side where the first sound would have been presented. It is important to note that subsequent repetitions of the bar were presented always in the same spatial position, without any further displacement. This starting physical inducer prevented the stochastic coupling between sound direction and bar illusory motion direction, with participants reporting a consistent illusory bar motion direction in the direction of the sound. The same physical inducer was present in both the AV and V experimental conditions. The number of repetitions of the flickering bar (5 or 6) together with the direction of the initial physical inducer (when the sound was either present or absent) allowed for a clear prediction for the direction of physical displacement produced by the SIVM illusion on each trial (see figure 1).

### Procedure and experimental design

Perceptual and oculomotor tasks were tested in separate blocks. Participants were required to undergo a calibration procedure for the eye tracker system at the start of each block. The trial started after acquiring a stable fixation for 500 ms (expressed as the mean fixation position during a 500 ms time window). All bar repetitions (5 or 6), except for the first physical inducer presentation (see “Apparatus and Stimuli”) were presented in the spatial position corresponding to the eccentricity condition value (i.e., 15, 16 or 17 degrees) of that trial with respect to the fixation position.

In the perceptual task, after the offset of the last repetition of the flickering bar participants were cued to press a left or right mouse button to report the last perceived movement direction of the bar by using their dominant hand. Participants were requested to press either the left button, if the bar was perceived to shift towards left, or the right button, if the bar was perceived to shift towards right. If no clear shift was perceived participants were requested to guess. In the visuo-motor task participants were instructed to move their eyes towards the last perceived position of the bar at the offset of the fixation point which occurred 50 ms before the offset of the last presented bar. A gaze contingent display paradigm was implemented to check whether participants correctly followed the instructions during the required time period. The trial was repeated whenever participants moved their eyes before the fixation offset, or before the presentation of the last bar in the perceptual task. This on-line gaze control was based on a confidence rectangle of 3×3 degrees visual angle around the initial fixation point on each trial. The experimental design comprised 12 different conditions: 3 eccentricity positions of the bar (15, 16, or 17 degree), 2 fixation positions (4 degrees to the left or right from the screen midline) and 2 flickering repetition values (5 or 6 repetitions). Within each response modality (i.e., each experimental block), these 12 conditions were repeated 4 times. Each block contained 48 trials. Overall, there were four blocks with a perceptual response and four blocks with visuomotor response in each session.

Each participant underwent V (visual) and AV (audio-visual) sessions on two different days. Both response modalities (i.e. perceptual and visuo-motor) were tested within the same day. Response modality and experimental conditions order were counter-balanced across participants. Before each experimental session of each day (perceptual and visuo-motor modality), participants underwent a training session of 12 randomly generated trials with the same conditions as the subsequent session. These training trials were discarded and not included in the analysis.

### Data analysis

For the perceptual task, responses were categorized as “far” and *“*close”. A response was categorized as *“*far” when the participant responded “leftward motion” with the fixation on the right side of the screen or responded “rightward motion” with the fixation on the left side of the screen. Responses were categorized as “close” otherwise. The mean proportion across participants of 'far' responses was computed for each *stimulus modality* condition (V and AV) and for each *expected response* condition (“far” and “close”). The expected response was computed based on the number of repetitions of the flickering bar (5 or 6 times) and the direction of the initial physical inducer. Consider, for example, the case in which a bar was shown on the left side of the screen with respect to fixation. If the final illusory motion was towards the right (the initial physical inducer shifted towards the left and the bar was presented for 5 repetitions), then such a trial was categorized as “close”. However, if the bar was shown on the right side of the screen and the illusory motion was to the right, then it would be classified as a ”far” trial (see Figure 1).

It is important to note that the “far” and “close” coding of trials holds either for the V (visual) or the AV (audio visual) conditions. In fact, even in the absence of the sound, the presence of the direction specific physical inducer allowed us to derive an expected perceptual as well as motor response, according to the presentation side and the number of bar repetitions.

For the visuo-motor task the distance between the eye movement landing position and the flickering bar position on the screen was computed for each trial and averaged across participants for each condition and each expected response, as in the perceptual task. This transformation was applied in order to obtain comparable values, independent of the actual bar eccentricity. Horizontal and vertical components of the eye movement were analyzed separately. During offline analysis, only trials in which the first eye movement was larger than 7 degrees of visual angle were considered valid (average mean of 85% of valid trials for across participants). A within-subjects ANOVA was performed on the results of the behavioral and the visuo-motor tasks. Linear regression and robust linear regression were adopted to test the magnitude of the effects in the different tasks and conditions.

## Results

**Figure 2:**
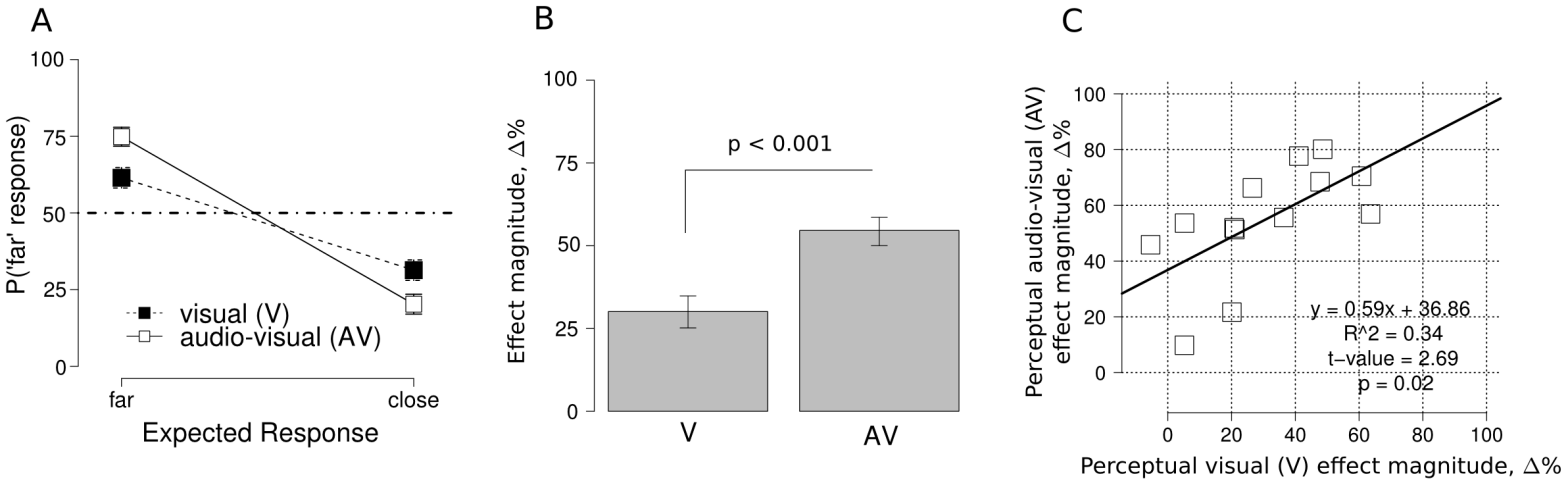
Results of the perception task. Panel A: the proportion of “far” responses for the two stimuli conditions (visual vs. audio-visual; V and AV) as a function of expected response. The steeper slope in the AV condition indicates a larger illusion effect (bars represent 2 SEM around the mean after normalization to remove between-subject variability [32]). Panel B: post hoc analysis representing effect magnitude in the V and AV condition. Magnitude in the latter condition is bigger than in the former, nonetheless V condition magnitude is consistently smaller but statistically different from chance (see results section; bars represent 2 SEM around the mean after normalization to remove between-subject variability [32]). Panel C: regression analysis in the perceptual task, the effect magnitude in the AV condition is positively correlated with magnitude in the V condition.

As expected, the sound-induced visual motion illusion influenced perception (Figure 2, panel A). Confirming that the SIVM illusion was consistently perceived in this experiment, an analysis of variance (ANOVA) on the proportion of “far” response in the perceptual task with factors *expected response* (“far” vs “close”) and *stimulus modality* (“V” vs. “AV”) showed a significant main effect of the latter variable in direction of the illusory movement (F (1,12) = 64.82, p<.001, η^2^_partial_ = 0.84). Importantly, a significant interaction between the two factors was found (F (1,12) = 23.69, p<.001, η^2^_partial_ = 0.68). In the perceptual task, the main effect of *stimulus modality* was not significant (F(1,12) = .01, *ns,* η^2^_partial_ = 0.02). To better understand the role of the sound in the AV and V conditions, the magnitude of the effect was computed for each participant and stimulus modality condition as the difference between the proportion of “far” responses for the different expected response conditions (Δ = p('far' | expected response  =  =  far) - p('far' | expected response  =  =  close), see figure 2 panel B). A paired two sample t-test showed that the magnitude in the AV and V conditions were statistically different (t(12) = 4.86, p<0.001). Notably, SIVM magnitude in the V condition, given only by the presence of the physical inducer, was smaller than the effect magnitude in AV condition. However, it is interesting to note that the illusion in the V condition was greater than zero (two-way, one sample t test, t(12) = 5.00, p<0.001). This indicates that although the mere presence of an initial physical inducer can drive some residual direction specific alternation, this effect is weaker without the accompanying sound, as can be seen in Figure 2 (panel B). This last result suggests that the presentation of the physical inducer can establish an alternating motion percept when position information is hard to extract reliably, as is the case for peripheral stimuli. Thus, we also report a novel visual illusion, in which a single offset perpetuates perceived alternating motion over repeated static flashes of the target. To our knowledge this is the first time that this effect has ever been reported.

Interestingly, a within subjects analysis showed that the magnitude of the effect in the V condition was correlated with the magnitude in the AV condition (see figure 2 panel C, linear regression, t(11) = 2.69, p<0.05, r^2^ = 0.34). In other words, participants who had a larger vision-only illusion also had a bigger audio-visual illusory perception of motion. It is important to note that the intercept parameter was significantly different from 0 (t(11) = 4.58, p<0.001). This strongly suggests a specific sound-induced effect since, in the theoretical case of magnitude effect equal to 0 in the visual (V) condition, the magnitude of the effect in the AV condition would still be different from zero.

**Figure 3:**
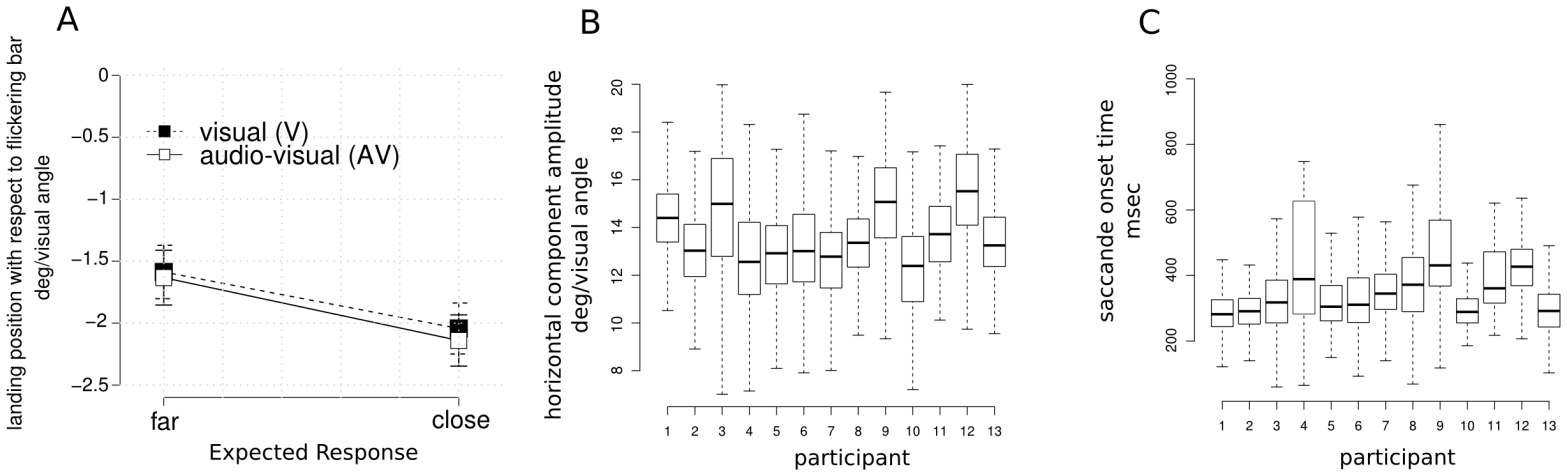
Visuo-motor modality ANOVA results. Panel A: average horizontal (x) component offset from flickering bar position (0 in the y axis) across experimental condition. Data shows a significant influence of expected response condition (bars represent 2 SEM around the mean after normalization to remove between-subject variability [32]). Panels B and C: Boxplots showing single participant distribution of horizontal component amplitude and saccade onset time across all conditions.

One of the main goals of the present study was to test whether the illusory percept of motion influenced saccadic landing positions. This was indeed the case. The effects of the experimental manipulations were confirmed by an ANOVA on the horizontal component of eye movements in the visuo-motor task with factors *expected response* (“far” vs. “close”) and *stimulus modality* (“V” vs. “AV”), showing a significant effect of *expected response* (F(1,12) = 18.09, p<.01, η^2^_partial_ = 0.60). Saccadic position was biased along the direction of the visual illusion (see Figure 3 panel A). That is, when the direction of the last repetition of the bar was perceived to move away from initial fixation, saccades were larger in magnitude and vice-versa.

Interestingly, neither a main effect of *stimulus modality* (A vs. AV, F(1,12)<1, *ns,* η^2^_partial_<0.01) nor interaction between *stimulus modality* and *expected response* emerged (F(1,12)<1, *ns,* η^2^_partial_ = 0.02). Thus, at the macro level, the visuo-motor illusion effect appeared to be comparable with and without the sound, in contrast to what was found with the perception task. The same analysis performed on the vertical component of saccadic eye movements did not yield significant results for any factors nor the interaction parameter.

**Figure 4:**
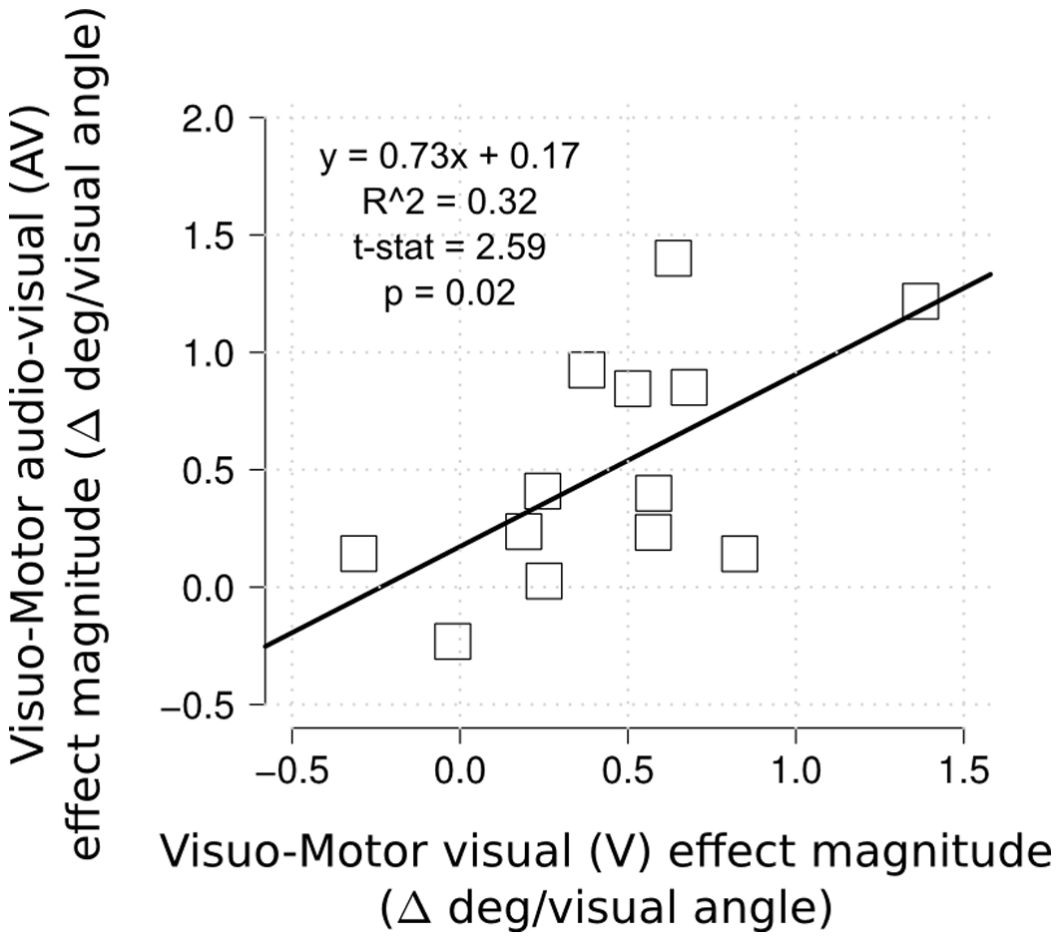
Analysis of individual subject performance in the different tasks and conditions. Data shows a strong correlation between the strength of the illusion in the two stimuli conditions (visual vs. audio-visual; V and AV) within the visuo-motor task.

The magnitude of the effect in the visuo-motor task was computed for each participant and condition as the difference between eye movement landing position and the flickering bar position distance, for the different expected response condition (Δ = distance(expected response  =  =  far) - distance(expected response  =  =  close), see Figure 4). As with the perceptual measurements, the magnitude of the effect in the AV condition was clearly correlated with the magnitude in the V condition (see figure 4, linear regression, t(11) = 2.59, p<0.05, r^2^ = 0.32). That is, participants who showed an effect of the illusion on saccadic landing positions showed similar effects with and without sound. In this case the intercept parameter did not reach significance.

Based on this pattern of results, one might hypothesize a possible dissociation between perception and action in our experiment. In fact, there was an added effect of the sound (AV vs. V conditions) in the perception but not in the oculomotor measures, supporting a weak interpretation of the two-visual system hypothesis. However, looking more closely at the data within subjects it becomes clear that the perceived illusion and the eye movement patterns were closely related.

**Figure 5:**
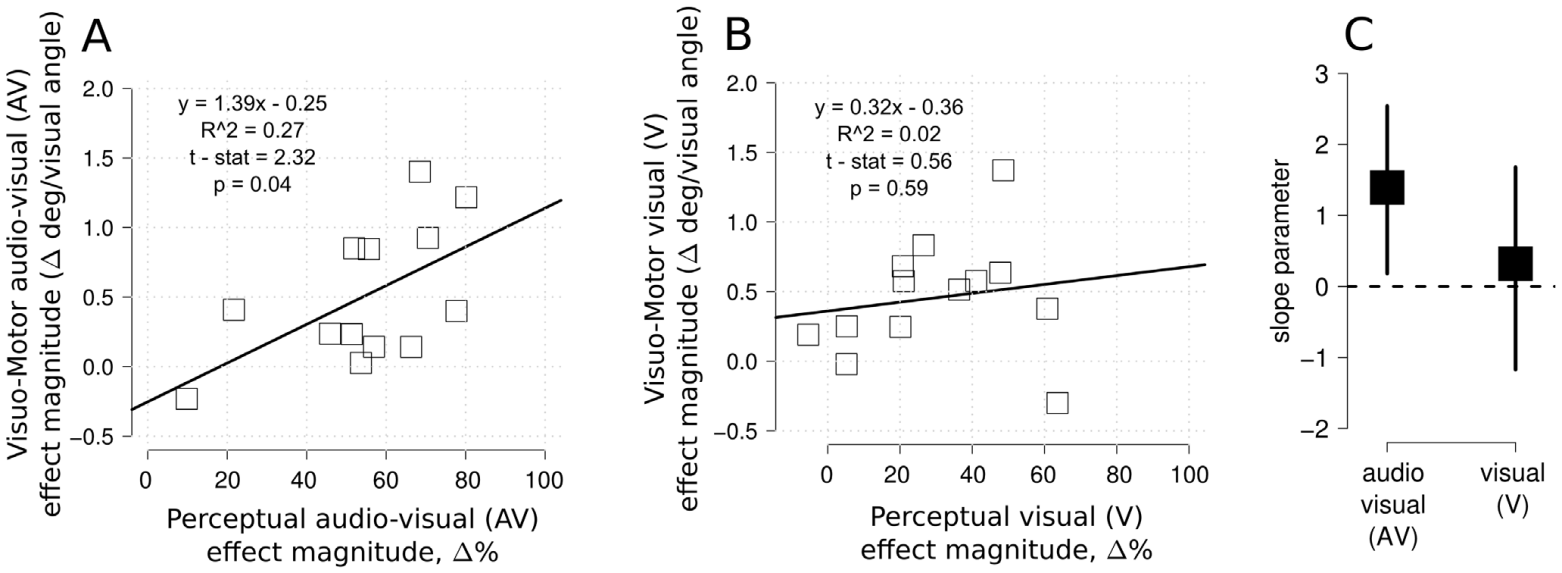
Analysis of individual subject performance in different tasks and conditions. Panel A: correlation between the visuo-motor and the perceptual response in the AV condition. Panel B: there was no correlation between the visuo-motor modality and perceptual modality in the visual condition (V). Panel C: 95% confidence interval bootstrapped slope parameter for the AV and V-only condition. Only in the former case the slope parameter was significantly above zero, whereas this was not the case in the V-only condition.

There was a significant linear relation across participants (r2 = .27, t(11) = 2.32, p<.05) between the magnitude of the illusion effect in the two tasks when the sound was present (figure 5 panel A). This result indicates that in the AV condition the magnitude of the illusory effect in the visuo-motor task increased along with the magnitude of the illusory effect in the perceptual task. Thus, it was possible to predict the amount of visuo-motor illusory effect from the magnitude of the perceptual illusion (and vice versa), supporting a close relation between perception and action systems. Critically, this relation was not present in the vision-only condition when analyzing magnitude effect across perceptual and visuo-motor modality (r2 = .02, t<1, ns, see figure 5 panel B). To test the role of influential values in each fit (audio-visual and vision-only) we ran a bootstrapping analysis (2000 resampled bootstrapping test) and reported the 95% percentile confidence interval on each slope parameter (figure 5 panel C). The slope parameter was significantly above zero only in the audio-visual illusion condition, whereas this was not the case for the visual-only condition.

To further test the difference between the audio-visual and visual-only condition we compared the two conditions in a single model, testing the effect of perceptual magnitude and sound condition on visuo-motor magnitude. Perceptual magnitude was treated as a continuous independent variable and sound condition as a dichotomous independent variable. Significance levels were tested with 95% confidence intervals in 2000 bootstrapping repetitions

**Figure 6:**
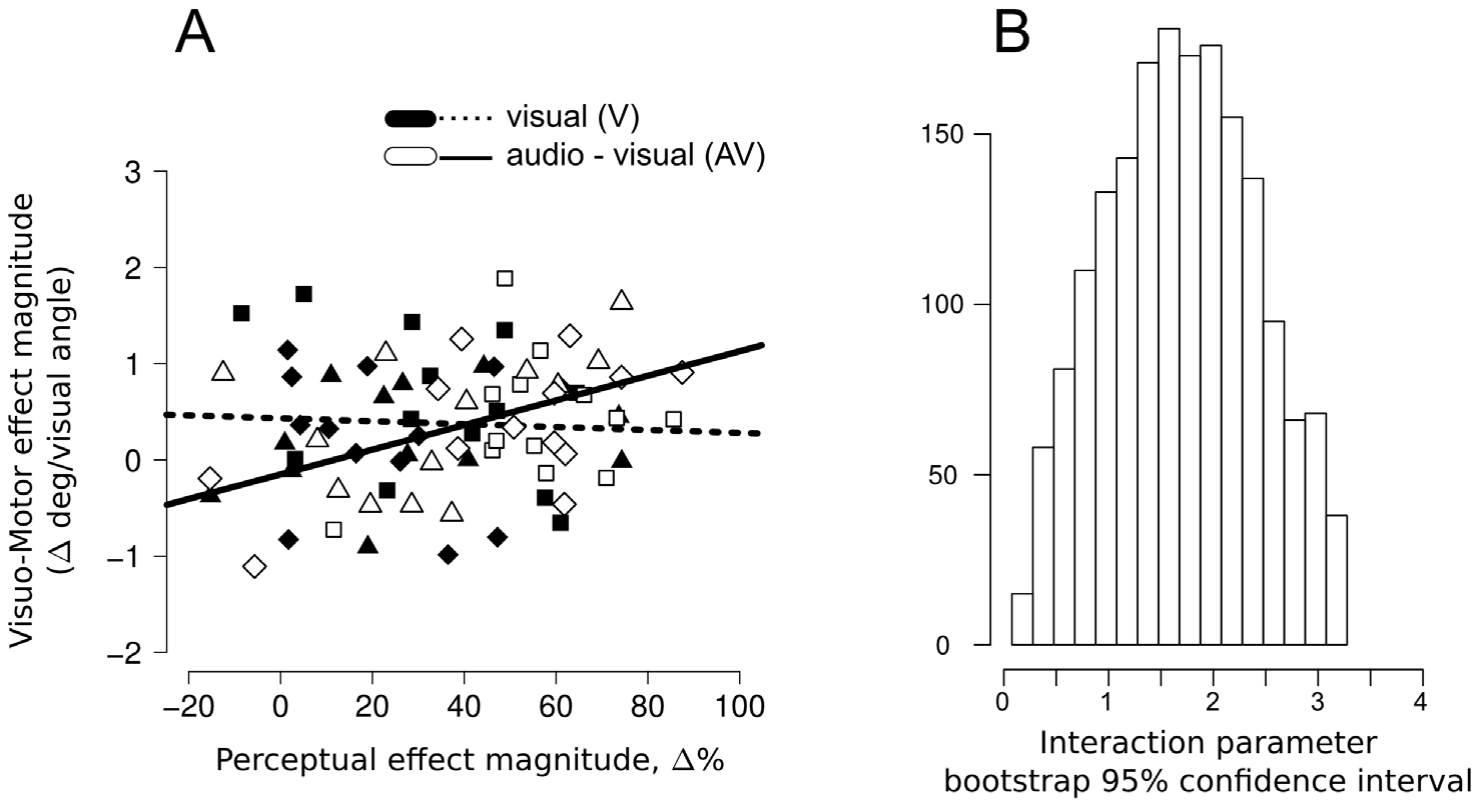
Bootstrapped robust linear regression links illusion magnitude in the perceptual and the visuo-motor domains. Panel A: perceptual – visuo-motor relation binned across the 3 eccentricities for each participant (squares  = 15 deg/vis angle, diamonds  = 16 deg/vis angle, triangles  = 17deg/vis angle); audio-visual condition (white symbols, thick line): y = 1.24x−0.19, t(37) = 2.90 (slope parameter, p<0.05 based on 2000 bootstrapped repetitions), r^2^ = 0.57; visual-only condition (black symbols, dotted line): y = −0.15x+0.48, t(37) = −0.28 (slope parameter, ns based on 2000 bootstrapped repetitions), r^2^ = 0.08. Panel B: 95% confidence interval of interaction parameter between audio-visual and visual-only condition suggesting how the slope in the audio-visual condition is significantly different from the visual only-condition (mean bootstrapped interaction parameter  = 1.58).

For each task we computed the magnitude on the illusory effect for each participant and level of eccentricity position of the bar (15, 16, or 17 degree of visual angle). There was a significant linear relation between the magnitude of the illusion effect in the two tasks when the sound was present (AV condition, r2 = .57, t(37) = 2.90, p<.05, robust linear regression, p value estimated by 2000 resampled bootstrapping sets, figure 6A thick line, white symbols) but not when the sound was absent (V condition, r2 = 0.08, t(37) = −0.28, ns, robust linear regression, p value estimated by 2000 resampled bootstrapping sets, figure 6A dotted line, black symbols).

Moreover, as can be seen in Figure 6B, there was a significant interaction between perceptual magnitude and sound, showing that the slope in the AV condition was steeper than in the V condition.

This result indicates that in the AV condition the magnitude of the illusory effect in the visuo-motor task increased along with the magnitude of the illusory effect in the perceptual task, supporting a close relation between perception and action systems. Thus, a careful examination of the results showed that, despite the difference at the macro level, there was still a close relationship between the two tasks at the level of individual participants. The influence SIVM on visuo-motor behavior rules out a strong interpretation of the two-visual system hypothesis. Moreover the overall pattern of results does not support a weak interpretation of the hypothesis, providing evidence in support of a shared representations between visuo-motor and perceptual systems.

In general, the illusion influenced the horizontal component of the saccade consistent with a perceived shift in position. However, on average, participants tended to perform slightly smaller saccades than requested (Figure 3, panel A). This is not surprising given the nature of the task and the large amplitude of the required saccades (16 degrees of visual angle on average), since undershoots are commonly reported in similar studies [14]. Participants performed “blind saccades” towards a target that disappeared 50 ms after the eye movement cue (see figure 1), which was extinguished by the time the eyes started to move (average saccade onset time across participants 336 ms, SD  = 80 ms).

## Discussion

The aim of this study was to test the link between action and perception by measuring whether oculomotor behaviour might be biased by the SIVM audio-visual illusion. Using a modified version of SIVM paradigm we were able to replicate the illusion in the perceptual judgment task. Moreover, we found a consistent effect of the SIVM illusion on saccade landing positions. The horizontal component of the eye movements towards a flickering bar perceived as shifting away from the initial fixation point was larger than when the bar was perceived as shifting towards the initial fixation point, and vice-versa. Thus, both perception and action were fooled by the illusion.

Interestingly, for both perceptual judgments and visuo-motor behaviour the mere presence of the physical inducer was sufficient to establish an alternating motion perception also in the absence of a coupled spatially specific sound. It is important to point out that the inducer stimulus was only presented at the beginning of the trial, either 2 or 2.5 seconds before the last bar in the sequence (for 5 and 6 repetitions conditions, respectively). Thus, the effect of the physical inducer persisted over time across subsequent repetitions of the stationary bar. To our knowledge this is the first time that this effect has been reported. This unexpected result has potential implications for studies of target localization in the far periphery [26] and requires further study. It has been shown that moving stimuli produce far reaching sub-threshold waves of activity in primary visual cortex spreading far ahead of the actual stimulus representation that “prepare” the cortex for an object’s putative trajectory [27]. In the far periphery, this anticipatory sub-threshold activity might be related to the established alternation observed here. The current findings add something new to those reported by Hidaka and colleagues [20] by showing that, in addition to a synchronous auditory inducer, also a single visual inducer can bias the subsequent perception of a static flashing stimulus so that it seems to move. One interesting question for future studies is whether the AV illusory motion and the visual inducer motion found in the present study reflect a shared mechanism (such as a shift in attention or preparatory activity in cortex for expected motion) or different modality-specific mechanisms. The correlation between audio-visual and visual-only induced motion illusions is suggestive of the possibility of a single mechanism.

More generally, the current pattern of results is relevant to the debate regarding potential dissociations between perception and action. Based on a simple comparison of whether the illusion manipulation (AV vs. V conditions) resulted in a significant difference or not between the two tasks, our results might at first seem to support a weak interpretation of the perception/action dissociation [11]. Indeed, the AV condition had a larger effect on perceptual judgments than the V condition, but no such difference was found on the eye movement landing position. However the correlation between the magnitude of the perceptual and the oculomotor effects in the AV but not the V condition (Figures 5C&6A) suggests a close coupling between action and perception systems. Thus, a more detailed analysis of the same data led to evidence for a common representation driving both the perceptual and the visuo-motor modality in the SIVM illusion. This idea was further supported by the robust linear regression approach in a single model (see figure 6B), showing a significant interaction between perceptual magnitude and sound, with the slope in the AV condition being steeper than the V condition. These findings resemble those obtained by Smeets & Brenner [28] in which the authors show how motion and location signals are processed independently, and these different sources of information are kept separated for both perception and action systems.

Another important aspect to be discussed is whether our findings are specific to the oculomotor system, rather than grasping or pointing hand movements as in many earlier studies. It has long been noted that saccades and attention are closely linked [29], [30]. Thus, the pattern of results found here might not hold for other sensory-motor systems such as those that subserve hand-related actions. Regarding the question of whether the oculo-motor effects found here are based entirely on attention shifts, it is notable that previous studies have shown reduced effects of visual illusions on saccade amplitudes with full attention [17]. In a study of Muller-Lyer illusion [17], it was reported that illusion effects on eye movement were largest for fast compared to longer saccadic latencies. Longer latencies result in a smaller illusion, suggesting that with sufficient time the oculomotor system was able to determine an accurate position of the target.

Overall, our results suggest that the spatial localization mechanisms involved in perceiving the flashing bar and targeting a saccade to that bar relied upon a shared neural representation. Moreover our data shows that the so-called sound-induced visual motion illusion can fool both perception, and eye movements, and that this can occur even without any sound at all based on the mere presence of a visual inducer. Such an inducer was able to establish a long lasting alternation in the periphery of the visual field. While the nature of shared representations between action and perception for a variety of features remains a matter of debate [11], [31], [3], the current findings provide support for a common mechanism in spatial localization.
